# Spiritual Inspiration of Village Cadres and Inclusive Innovation of Bricolage in Rural Autonomy in China

**DOI:** 10.3389/fpsyg.2021.617838

**Published:** 2021-08-04

**Authors:** Qing Zhao, Mei Yu, Liangcan Liu, Binghan Li, Li Feng

**Affiliations:** ^1^School of Business Administration, Guizhou University of Finance and Economic, Guiyang, China; ^2^School of Finance, Zhongnan University of Economics and law, Wuhan, China; ^3^School of Politics and Law, Huanggang Normal University, Huanggang, China

**Keywords:** Chinese village, spiritual inspiration, Inclusive innovation, bricolage, affective commitment, resilience

## Abstract

In the rural autonomous context of China, inclusive innovation of bricolage is a widespread phenomenon. However, the existing research rarely considers villagers as the main actors of innovation. In a research survey of 23 provinces in China, we studied the internal mechanism of inclusive innovation of bricolage. We found that the spiritual inspiration of the village cadres is positively related to inclusive innovation of bricolage behavior of villagers. Moreover, the results revealed that affective commitment to the village of the villagers plays a mediating role in the relationship between the spiritual inspiration of the village cadres and inclusive innovation of bricolage behavior of villagers. In addition, our findings reveal that resilience of villagers plays a moderating role in the relationship between the spiritual inspiration of the village cadres and inclusive innovation of bricolage behavior of villagers.

## Introduction

In China, under the influence of the collective economy, clan culture, official, and gentry culture (Deng, 2012), and the change of population structure, the surroundings of rural autonomy are different and more complex than in the United States, Britain, and France in Western Europe, as well as Japan and South Korea in East Asia (Nie, 2012; Shen and Liu, 2016). The governance model of urban and rural areas in Chinese society is dualistic, which is reflected in the fact that the street, the smallest governing unit of a city in China, is subordinate to the government, while the village committee and the village party branch—the smallest governing unit of a village in China—are not nominally subordinate to the government. The village committee and the village party branch are autonomous rural units elected by villagers rather than appointed by the government, and their leaders are often members of the rural elite with local prestige and influence. The collective economy is represented by rural cooperatives and by traditional rural clans. These groups are important in maintaining rural autonomy in China and in ensuring the stability of the development of the village. At the same time, rural autonomy of China is inextricably linked with the government. In keeping with the traditional culture of officials and nobility, the heads of rural governance institutions of China are often hereditary leaders; self-identity of quasi-official status is common among the heads of rural autonomous institutions (Wang and Chen, 2011). Furthermore, due to the large-scale outflow of rural population resulting in the lack of rural governance elites, the rural management of the government is not only constrained by a budget but also affected by a shortage of talent. As a result, it has to rely on autonomous rural institutions (Wang C, 2020). Therefore, to a certain extent, autonomous rural institutions of China have become extensions of local governments insofar as they share aspects of government functions.

In 2015, China started to implement the influential targeted poverty reduction initiative, focusing on the vast rural areas. The initiative is a comprehensive, sustainable, nationwide strategic plan aimed at achieving a prosperous society in a holistic way. In this unprecedented initiative, enhancing innovation is an important means to increase the income of the poor. Therefore, the extent to which Chinese villagers can use the resources provided by the government and society to innovate their production mode, production technology, and business model largely determines the actual effect of the targeted poverty reduction initiative. Fortunately, there is evidence indicating that a large number of innovative behaviors of Chinese farmers are involved in production, operation, and entrepreneurship (Tu and Chen, 2018; Yang, 2020; Zhong and Kong, 2020; Pan, 2021) in what we call the inclusive innovation of bricolage (defined as integrating available and usable resources in a piecemeal way, with the advantage of involving and benefiting the poor) (You et al., 2021). The topic of this study is this bottom-up inclusive innovation behavior in complex rural autonomous surroundings of China, how it is influenced by the spiritual inspiration (SI) of the village cadres who have a close relationship with the villagers, how it works through the unique spiritual connection of Chinese villagers, and how it is related to the personal characteristics of the villagers.

There are many early studies on the topic of rural autonomy in China, which focus on the rural political system itself. Based on research on rural autonomy system of China (Jean, 1989, 1996; Lawrence, 1994; O'Brien, 1994, 2001; Jean and Rozelle, 2000; Brandtstädter and Schubert, 2005; Manion, 2006; Zhao, 2006), the issue of incentive and performance in rural autonomy of China has become a hot topic in recent years. However, some of these studies only put forward theories and did not carry out empirical research (Liu, 2018; Wang, 2018; Wang C, 2020; Zhang and Hua, 2018). Some of them based their findings on the analysis of a small number of cases (Zhao et al., 2013; Lu, 2018; Gu, 2019; Wu, 2020). Few scholars collected extensive sample data for empirical analysis (Chen et al., 2014; Yang and He, 2019).

Existing research on inclusive innovation mainly takes enterprises as the main actors in innovation, mostly from the perspective of enterprises (Chikweche and Fletcher, 2010; Yunus et al., 2010; Hens, 2012; Prahalad, 2012; Wooder and Baker, 2012; Zhang et al., 2012). Few research studies are taken from the perspective of the lowest ranking groups (Anderson and Markides, 2007; Tao et al., 2019). There is no research taking the lowest group as the innovation actors and taking the perspective of the lowest group. However, the concept of inclusiveness means not only the sharing of achievement but also the equality of opportunities. Therefore, if we only study inclusive innovation by taking enterprises as the main actors, the concept of inclusive innovation is not fully covered because it does not cover the innovation behavior of the lowest ranking groups. Therefore, we take Chinese villagers, the groups that need to be included, as the main actors in our exploration of inclusive innovation.

In Chinese villages, village cadres have a significant influence on the behavior of villagers because of their authority (Yang and He, 2019). Many research studies have confirmed that the spiritual motivation of leaders in enterprises can influence the IB of their followers (or subordinates) (Dennis and Ashmos, 2005; Kim and Park, 2015; Li, 2018; Deng et al., 2019; Qi et al., 2019; Giorgi et al., 2020). Specifically, the SI of village cadres of China has a unique influence on IB of the villagers. As a semiprofessional leader wandering through the gray area of administrative governance (Shu, 2020), to a certain extent, village cadres play the role of parents in rural grassroots (Di, 2019). The relationship between village cadres and villagers is not a formal relationship between leaders and subordinates; it is more like the relationship between tutors and students. Therefore, in the context of the education level, moral level, and working ability of villagers (Yang and He, 2019), SI of the village cadres plays a prominent role in encouraging villagers to become rich and in promoting the development of villages (Hu, 2019).

Based on the theory of spiritual leadership and charismatic leadership, the existing studies have conducted empirical research on how the authority spirit, team spirit, entrepreneurial passion, and other attributes of village cadres affect villagers. He and Akuzhiko (2006) proposed that the village cadres are the elite of the rural front-line administrators, the models and the spiritual source for the villagers; Li (2018) revealed that SI of the village cadres helps to generate affective commitment (AC) to the village within the village. Bengt and Morten (2000) found that SI of the village cadres can help villagers to avoid the loss of innovation resources, increase their enthusiasm (motivation) for innovation, promote the realization of innovation, and ultimately enhance the survival and development ability of the village. In analyzing the authority of the village leaders and their overall influence on rural development, Yang and He (2019) point out a particular relationship between the innovative behavior (IB) of the villagers and the knowledge and charisma of the village head. However, existing research cannot fully explain the cognitive and behavioral logic of village cadres and villagers. Village cadres are not formal leaders, and villagers are not employees in an enterprise. The concept of SI proposed by Fredrickson and Anderson (1999) is an important part of the theory of spiritual leadership and charismatic leadership. Fry (2003) and Duchon and Plowman (2005) conducted an in-depth discussion of semiprofessional leadership. Our work is based on their research. At present, there are many studies on the results or effects of SI, mainly considering organizational performance, job satisfaction, occupational satisfaction, environmental behavior, mental health, empowerment, and altruism. In contrast, research on the IB of employees is relatively scarce (Oh and Wang, 2020). Moreover, there is a lack of research on the innovation behavior of farmers (as an employee in a sense), and the relevant policies on the innovation of villagers are rarely mentioned (Li, 2007; Chen, 2014). Therefore, there is a need to carry out more discussion.

In the pre-survey, we found that SI of the village cadres is mainly conveyed through collective events, such as meetings and gatherings in the village rather than through private contacts between village cadres and villagers. Moreover, the response of the villagers to this kind of SI is often strengthened in the context of the village. There can be a positive influence of the clan and the other villagers. Therefore, we considered that the influence of SI of the village cadres on the behavior of villagers is largely based on the village organization. Consequently, we examined the mediating role of AC, that is, the degree of emotional attachment, identification, and involvement of individuals in the organizations (Meyer and Allen, 1991), and the impact of SI of the village cadres on inclusive innovation of bricolage of villagers. At the same time, we found that the villagers who achieve most in innovation and entrepreneurship activities are often those who can adjust and adapt quickly in the face of change, adversity, or interference (Grotberg, 2005). Therefore, we investigated the moderating effect of resilience of the villagers in the process of SI of the village cadres, influencing IB of the villagers.

To clarify the relationship between SI of the village cadres and the bricolagical-inclusive IB of villagers in complex autonomous rural surroundings of China, we conducted an empirical study based on a survey of farmers from 23 provinces in China. First, we identified and investigated the positive influence of SI of the village cadres on IB of villagers. Second, we revealed the mediating role of AC of villagers in the influence of SI of village cadres on IB of villagers. Finally, we revealed that the resilience of the villagers plays a moderating role in the influence of SI of the village cadres on IB of the villagers.

An important contribution of our research is the identification of the moderating role of personal psychological traits in the influence of leader motivation on IB of followers. Previous literature only found that external forces or personal perception plays moderating roles. We also found that AC plays a mediating role in the influence of motivation on IB. In the previous literature on the influence of leader motivation on IB of followers (employees), AC has been considered as a moderator, but other possible effects have not been studied (Li, 2018). In addition, we expand the research literature in the organizational behavior field on the motivation of leaders as an influence on IB. In other words, we focus on the incentive value of rural cadres for farmers rather than the incentive value of entrepreneurs for followers (or subordinates).

Our research also contributes to rural governance literature by exploring the incentives for and performance of rural autonomy in China from the perspective of inclusive innovation of bricolage. Moreover, it contributes to the field of inclusive innovation, taking the bottom group as the main actors of innovation. Specifically, we highlight equal opportunity in the concept of inclusiveness in empirical research, which is different from the previous research, taking enterprises as the main actors of innovation and only emphasizing shared achievement.

The structure of this study is as follows: first, the logical relationship among the four components of SI of village cadres, IB, AC, and resilience of villagers is theoretically revealed; second, the data, variables, and methodology are introduced; third, the hypotheses are tested, using the structural equation model, and the results are discussed; finally, the theoretical findings and practical implications are summarized.

## Literature Review and Hypotheses

Village cadres are members of a village whose encouragement to and mobilization of villagers are regarded as critical factors in cognition and behavior remodeling of villagers. Researchers have formed different research paradigms around this theme. Scholars mainly discussed system repression, power domination, interest exchange, asylum, and spatial dependence (Di, 2019). With the continued expansion of quantitative research in recent years, more attention has been paid to the in-depth analysis of the mechanism of the “cadre–member” system from a micro perspective, such as the influence of SI on IB and the mediating factors in this system.

## Spiritual Inspiration and Innovative Behavior

Amabile et al. (1996) pointed out that all IBs begin with ideological innovation, and SI is one of the driving factors. According to the component theory of organizational innovation, in addition to SI, team support, corporate belief or vision, care, and material incentives are also the driving factors of innovation in the members of a team (Deng et al., 2019). Some studies found that unless villages have organizational support, collaborative care, and material incentives, IB will not occur. When village-level organizations are highly developed, reflection of the villagers in response to SI of village cadres is more prominent. The reasons are as follows: first, village cadres are neither civil servants nor managers nor law enforcers (Tang, 2001). In the absence of administrative resources, SI is an inevitable choice. Second, under the top-down administrative pressure, village cadres must take positive measures to complete the tasks issued by the government. Third, the traditional climate of rural regulations and compliance with authority makes sense of elite responsibility (Fei, 1948; Huang, 1986). The vague role positioning of state agents jointly promotes the self-identity and stronger self-efficacy of village cadres.

IB of Villagers can be attributed to any progressive change in traditional practices (Li and Xiang, 2010), such as the progressive change in agricultural production technology and the management mode. Researchers have not studied this subject thoroughly; the significance and the value of such research have been questioned. Although IB of farmers is a reality, some research studies indicate that it is still in an embryonic stage (Chen, 2014; Peng et al., 2016). However, in answer to the call for IB of villagers, relevant policies have been gradually improved, and academic discussions have deepened. In 2013, the report of the 18th National Congress of the Chinese Communist Party and the No.1 Central Document pointed out that initiatives of farmers must be promoted across the country. Some international conferences and influential journals have also begun to pay attention to innovation of farmers, as seen in Dolinska and D'Aquino (2016), Chindime et al. (2016), and Kopytko (2019). Research by Chinese scholars, such as Li (2013), is also highly significant.

Discussions about the relationship between village cadres and villagers often appears in discussions of the theory of village governance. Di (2019) pointed out that, as a particular agent between state administration and the villagers, the village head/village branch secretary has a critical impact on morale of villagers. Yang and He (2019) pointed out that the mobilization of administrative resources of the village head, the encouragement of ideological and public opinion, the shaping of cultural atmosphere, the support of policies and systems, and the protection of intellectual property rights could effectively promote innovation motivation and behavior of villagers. These studies (including the results of empirical and case studies) are of groundbreaking significance, but they ignore the unique status of village cadres as semiprofessional leaders. Given this, we focus on the influencing factors within the operational region of village cadres and discuss the significance and rationale of their SI on innovation behavior of villagers. This focus is in line with the original intention of Zhao et al. (2013), who found that the quality improvement of village cadres creates appropriate circumstances for innovation, thereby affecting the consciousness and behavior villagers on a spiritual level and ultimately promoting the process of rural socialization.

Based on the above research, this paper holds that the SI of the village cadres, as non-professional leaders, positively impacts the IB of the villagers.

H1: The SI of village cadres promotes IB of villagers.

## The Mediating Role of Affective Commitment Between Spiritual Inspiration and the Innovative Behavior of Village Cadres

We suppose that the impact of the SI of the village cadres on IB of villagers IB is affected by AC of the villagers to the village. Many scholars, such as Meyer and Allen (1991) and Meyer et al. (1993), have noticed the influence of AC on innovation behavior. According to them, the concept of AC involves the psychological relationship and performance between individuals and organizations. Judge and Kammeyer-Mueller (2012) further extended the scope of this concept, arguing that it also includes identification, participation, and emotional dependence of individuals on an organization, reflecting the degree of intimacy between individuals and organizations. Li (2018) supposed that AC of individuals can promote their innovation motivation and behaviors. AC is an aspect of team entrepreneurship passion theory and transformational leadership theory, whereby the mediating role of the spirit of the leader (including the atmosphere created) and innovation behavior of individuals is tested (Carmeli, 2005; Kark and Carmeli, 2009). The current popular team differential climate theory debate also attaches great importance to the mediating role of AC. Wang Q (2020) tested the mediating effect of emotional commitment between differential climate perception and individual innovation behavior. Many scholars suggest that self-efficacy should be adopted regarding the choice of a mediating variable (or a construct). Self-efficacy is the perception and subjective assessment of whether one can complete the work. In the pretest of the questionnaire survey, we found that this variable and IB scale items of the villagers confused the subjective judgment of the villagers, so we removed this variable. Although the above conclusions are drawn from the studies of different organizations, they do not affect the hypothesis whereby AC is used as a mediating variable in this study. Because of this, we propose hypothesis 2.

H2: AC plays a mediating role in the positive impact of SI of village cadres on IB of villagers.

## The Moderating Effect of Resilience

The SI of the village cadres can eventually arouse IB of villagers. There is also an essential moderating mechanism factor, personal resilience of villagers (such as perseverance, firmness, etc.). Gao and Bi (2009) pointed out that village cadres play an essential role in promoting “agricultural efficiency, income of farmers and rural development,” which is mainly reflected in guidance and mobilization of village cadres through SI. Eventually, this is transformed into the IB of the villagers (Li and Xiang, 2010). What is the internal influence mechanism of encouragement of village cadres on IB of villagers? Few studies have discussed this. Theoretically speaking, innovation behavior of individuals at the micro level is related to their innovation consciousness and their resilience (Hao et al., 2020). According to human ethology and social psychology, resilience is the dynamic ability to overcome setbacks and adapt to changes (Martin, 2012; Eicher et al., 2015), and it is also regarded as inspiring confidence of people in their careers. It is only when villagers are optimistic about their jobs that the SI of the village cadres can be transformed effectively into IB of villagers. According to organizational behavior theory, resilience is a more appropriate moderating variable (Gong et al., 2019). This paper argues that the impact of SI of village cadres on innovation behavior of villagers is strengthened or weakened by resilience of the villagers. Therefore, the following hypothesis is obtained.

H3: Resilience plays a moderating role in the positive impact of SI of village cadres on IB of villagers.

Some scholars point out that resilience often plays a positive moderating role between personal AC and innovation behavior. Bullough et al. (2014) examined the relationship between the perceptions of individuals of risk, self-efficacy, resilience, and entrepreneurial intention. They tested the positive moderating role of resilience in self-efficacy and AC in entrepreneurial behavior. We added a moderated-mediating model to test the moderating effect of resilience on the mediating variable of AC from the perspective of the impact mechanism of AC on IB of villagers. We derived a new hypothesis.

H4: Resilience moderates the mediating effect of AC in the impact of SI of village cadres on IB of villagers.

**Figure 1:**
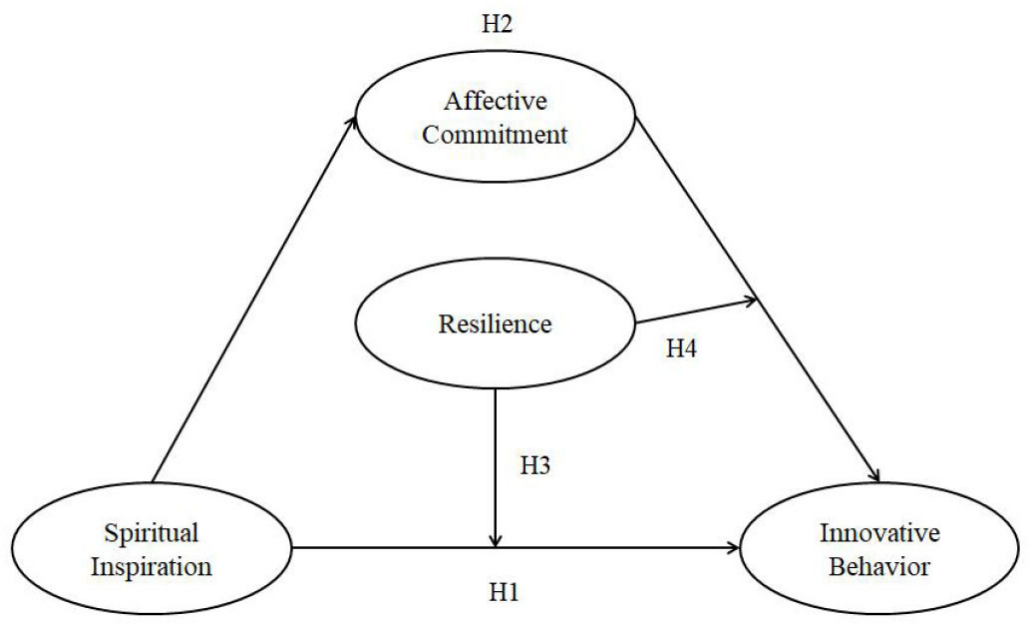
Research model.

The conceptual logic models in Figure 1 show the relationship of the four hypotheses.

## Methodology

### Sample Source

The sample for this study comes from the customers of a large fertilizer enterprise's distribution system, involving bulk blend fertilizer centers (BBF), with 46 distribution terminals in 23 provinces of China. We required each BBF center to randomly select 10 people from the villages they serve, and if the number was ≤10, all of the people were selected. Then a customer was randomly selected from each village, and a survey list of 320 farmers was obtained. In the first round of the survey, we asked the staff at the BBF center to issue 160 questionnaires. In the second round of the survey, we asked a group of college students who were participating in a holiday social practice project to administer 160 questionnaires. Finally, we had 274 valid questionnaires after eliminating the questionnaires with missing items or with obvious logical conflicts. The descriptive statistical results of the samples are shown in Table 1.

**Table 1:** Respondent demographics (*N* = 274).

**Items**	**Classification**	**Sample amount**	**Percentage (%)**
Gender	Male	147	30%
	Female	56	70%
Age	≤ 18	10	4.0%
	19–30	64	40.0%
	31–50	90	50.0%
	≥51	32	6.0%
Education	Primary school and below	5	19%
	Junior middle school	80	34%
	High school	65	43%
	University and above	16	4%
Number of children	≤ 1	31	69%
	2	42	16%
	3	75	11%
	≥4	23	4%
Marital status	Yes	149	69%
	No	54	31%
Party member	Yes	170	16%
	No	33	84%
Cooperative member	Yes	156	14%
	No	25	86%

As shown in Table 1, the majority of the participants were women, accounting for 70%. Of the individuals surveyed, 94% were below 50 years old, 96% had a high school education or lower, and the remainder had graduate education or above. Most of the participants were married (69%), 85% had fewer than two children, 84% were not party members, and 86% were not members of rural cooperatives.

### Measurement of Variables

A scale with good reliability and validity was used as the measurement tool. In line with Karasz and Singelis (2009), standard translation and two-way back translation were used to ensure that the Chinese and English versions of the scale had the same meaning. The village cadres helped to optimize the Chinese expressions used in the questionnaire to make them align with the rural reality and to ensure that the interviewees could understand and complete the questionnaire. Twenty-one items were integrated into the questionnaire, and a 1–7 Likert scale, as recommended by Bollen (1989), was used for propensity score evaluation, where 1 represented total disagreement, and 7 represented full agreement See Appendix 1.

The dependent variable “IB of villagers” was mainly based on the classical scale of individual innovation, developed by Scott and Bruce (1994), including “I will actively look for new technologies to improve labor efficiency,” “I will think about new ideas,” “I will introduce my new ideas to others,” “I will raise money for new ideas,” and “I will turn new ideas into reality.” The Cronbach's α of the scale was 0.897.

The independent variable “SI of village cadres” was based on the maturity scale developed by Fry (2003) (Cronbach's α value was 0.876). The scale consisted of three dimensions: vision, altruistic love, and belief or faith, with a total of 20 items. In line with the reality of rural China, we improved the item localization text and selected the item closest to the SI. The representative items included “I am confident in fulfilling the goals set by the village cadres,” “I always try my best to do a good job, because I have faith in the village collective leadership and the village cadres,” “I set a high goal for my own work, because I am confident in the village collective leadership and hope that the villagers can succeed,” “Our village collective leadership is trustworthy and always serves the villagers,” and “The village collective leadership really cares about the farmers.” The Cronbach's α of the scale was 0.948.

We used the expression “village collective leadership” in the items. Chinese rural areas are deeply influenced by the traditional culture of officials and gentry. Even if village cadres are not real officials, they still have quasi-official status in the eyes of villagers. In the official system of China, the top leader has considerable power, and organizational decision-making is often seen as a strong reflection of the personal will of the top leader. The same is true in villages. The spirit of village cadres depends on the village collective, and the decision-making of village collective leadership is regarded as reflecting the will of village cadres. To some extent, it can be said that the village cadres are the representatives of the village collective leadership, and the decision-making of the village collective leadership is regarded as the projection of the will of the village cadres. Therefore, the use of village leaders as the subject, in the context of the rural areas of China, still could measure the views of the villagers on village cadres accurately. So why did we not use village cadres as the subject directly? Because behaviors such as “serving” and “caring” are often not done by the village cadres themselves. They assign tasks to their subordinates or entrust other villagers to perform them on their behalf. If the behavior subjects in the items description were the village cadres themselves, it might make the villagers misunderstand.

The mediating variable AC was adopted according to the AC subscale of the organizational commitment maturity scale developed by Meyer et al. (1993) (Cronbach's α value was 0.86). The words used in the statements were localized, including “I am willing to work in the village in my spare time,” “I take issues of the village collective as my own,” “I have a strong sense of belonging to the village collective,” and “I want to be a member of the village collective.” The Cronbach's α of the scale was 0.899.

The maturity scale developed by Campbell-Sills and Stein (2007) was used to measure the moderator (bidirectional) “resilience.” The representative items were “I can adapt to changes,” “ I can always stay optimistic when encountering difficulties,” “I can continue to concentrate on my work under pressure,” “I can cheer up after being sick, injured, or experiencing other difficulties,” “Being under pressure makes me stronger,” “I think I can achieve my goal even if there are obstacles,” and “I can deal with unhappiness or other difficulties.” The Cronbach's α of the scale was 0.910.

We used the research results of Chen et al. (2013) to select the control variables. We used variables that influence innovation behavior, such as age and education. Song et al. (2013) believe that innovation behavior of farmers cannot be separated from their attitudes toward agricultural cooperatives. Therefore, we used whether the farmer was a member of a rural cooperative or not as a control variable. Yang and He (2019) believe that identity of Party members is an essential influencing factor, and this was also set as a control variable. This study also set gender, marital status, and number of children as control variables.

### Common Method Variance

The SI, innovation, AC, and resilience questionnaire were all completed by the villagers themselves, and this may have given rise to common method variance (CMV). Harman's single factor method and the correlation coefficient method were used to test the two constructs. Harman's single factor method is a non-rotating exploratory factor analysis of latent variable items. If an independent factor is precipitated or the variance explanation percentage of the first factor is more than 50%, it indicates severe CMV. In this study, there was no independent common factor separated. The variance interpretation rate of the first factor was 48.655%, below the recommended standard of 0.5 of Joseph et al. (1995), indicating that the common method deviation problem was not significant. If the correlation coefficient between constructs is higher than the recommended standard of 0.9, there is a higher CMV. As shown in Table 2, the maximum correlation coefficient was 0.674, which is far lower than 0.9. The CMV was within the acceptable range, indicating that there was no severe CMV.

**Table 2:** Descriptive statistics and correlation analysis (*N* = 203).

**Variable**	**M**	**SD**	**1**	**2**	**3**	**4**	**5**	**6**	**7**	**8**	**9**	**10**	**11**
Gender	1.701	0.459	–										
Age	2.277	0.632	−0.168^**^	–									
Education	2.310	0.818	0.024	−0.231^**^	–								
Number of children	1.518	0.882	−0.077	0.635^**^	−0.234^**^	–							
Party member	1.836	0.371	−0.053	0.023	−0.085	0.037	–						
Cooperative member	1.861	0.346	0.038	−0.259^**^	0.010	−0.256^**^	0.050	–					
Marital status	1.693	0.462	0.049	−0.699^**^	0.233^**^	−0.634^**^	−0.038	0.168^**^	–				
SI	3.431	1.360	−0.150^*^	0.056	0.050	0.034	−0.004	−0.068	−0.031	(0.949)			
IB	3.214	1.026	0.056	0.062	0.020	0.011	0.002	−0.061	−0.027	0.498^**^	(0.901)		
AC	3.287	1.191	−0.087	0.057	0.047	0.089	0.065	−0.070	−0.033	0.674^**^	0.664^**^	(0.900)	
ENR	2.847	0.947	−0.029	0.025	0.083	0.036	0.088	−0.026	0.005	0.397^**^	0.512^**^	0.565^**^	(0.913)

*** *p < 0.01*,

** *p < 0.05*,

* *p < 0.1. In brackets are Cronbach's α values of corresponding variables*.

### Descriptive Statistics and Correlation Analysis

Table 2 shows the mean, standard deviation, correlation coefficient, and square root of AVE value of each variable. The SI of village cadres was significantly positively correlated with IB of villagers (β = 0.498, *p* < 0.05) and AC (β = 0.674, *p* < 0.05). The AC was also significantly positively correlated with innovation behavior (β = 0.664, *p* < 0.05). Accordingly, H1 was preliminarily verified.

## Results

### Reliability and Validity Analysis

The reliability and the validity of the questionnaire were tested, as shown in Table 3. The combined reliability (CR) of the four variables of SI, IB, AC, and resilience was >0.9, which was higher than the recommended criteria of Fornell and Larcker (1981) (>0.6). The CR of the questionnaire met the requirements. The convergence validity of the questionnaire was tested by measuring mean-variance extraction (AVE). In Table 3, the load factors and AVE values of the potential variables are >0.6, respectively, which met the recommendations of Fornell and Larcker (1981) on AVE standard value (>0.5), indicating that the scale has good convergence validity. Simultaneously, the square root of the AVE on the diagonal is greater than the direct correlation coefficient of each potential variable in the same row or column, reflecting that discriminant validity of each scale also met the statistical requirements.

**Table 3:** Reliability and validity analysis.

**Dim**.	**Item**	**Factor loading**	**Composite reliability**	**Convergent validity**	**Discriminant validity**			
			**CR**	**AVE**	**SI**	**IB**	**AC**	**ENR**
SI	6	0.780–0.929	0.949	0.755	**0.869**			
IB	5	0.644–0.897	0.901	0.648	0.49	**0.805**		
AC	4	0.779–0.910	0.9	0.693	0.723	0.690	**0.832**	
ENR	6	0.734–0.866	0.913	0.636	0.410	0.529	0.608	**0.797**

*CR, composite reliability; AVE, average valiance extracted; the main diagonal number is the square root of AVE, displayed in bold*.

Construct validity was tested through confirmative factor analysis. The results, as shown in Table 4, indicate that the factor load of each factor in the four-factor model reached a significant level of 0.05, and there was no inappropriate solution. All the fitting indexes (X^2^/DF = 2.3; SRMR = 0.051; RMSEA = 0.069; CFI = 0.950; TLI = 0.942) met the recommended standard of Wu (2017), indicating that several constructs involved in this study have good structural validity. Simultaneously, compared with other nested models, such as the three-factor, two-factor, and single-factor, the quartet model has higher goodness of fit, which indicates that the model with four variables is the best.

**Table 4:** Construct validity analysis.

**Model**	***X*^**2**^**	**DF**	***X*^**2**^/DF**	**Δ X^**2**^ (ΔDF)**	**CFI**	**TLI**	**RMSEA**	**SRMR**
Four-factor model	420.825	183	2.300		0.950	0.942	0.069	0.051
Three-factor model a	1,110.411	186	5.970	689.586 (3)^***^	0.805	0.780	0.135	0.122
Three-factor model b	822.535	186	4.422	401.710 (3)^***^	0.866	0.848	0.112	0.099
Three-factor model c	1,314.158	186	7.065	893.333 (3)^***^	0.762	0.731	0.149	0.147
Three-factor model d	742.322	186	3.991	321.497 (3)^***^	0.883	0.867	0.104	0.063
Three-factor model e	1,010.914	186	5.435	590.089 (3)^***^	0.826	0.803	0.127	0.091
Three-factor model f	918.296	186	4.937	497.471 (3)^***^	0.845	0.826	0.120	0.097
Two-factor model a	1,601.039	188	8.516	1,180.214 (5)^***^	0.702	0.667	0.166	0.153
Two-factor model b	1,406.687	188	7.482	985.862 (5)^***^	0.743	0.713	0.154	0.129
Two-factor model c	1,637.168	188	8.708	1,216.343 (5)^***^	0.694	0.658	0.168	0.151
One-factor model	2,088.659	189	11.051	1,667.834 (6)^***^	0.599	0.555	0.192	0.129

*The four-factor model: SI, IB, AC, and resilience; the three-factor model a: SI and IB are combined into one factor; the three-factor model b: SI and AC are combined into one factor; the three-factor model c: the fine three-factor model d: IB and AC are combined into one factor; the three-factor model e: innovation behavior and resilience are combined into one factor; the three-factor model f: AC and resilience are combined into one factor; the two-factor model a: SI and IB are combined into one factor, AC and resilience are combined into one factor, the two-factor model b: SI and AC are combined into one factor, IB and resilience are combined into one factor; the two-factor model c: SI and resilience are combined into one factor, IB and AC are combined into one factor; the single-factor model: all variables are combined into one factor*.

*** *P < 0.001*.

### Main Effect and Mediating Effect Test

Regression analysis is applied to test the relationship among the SI, AC, and IB of village cadres. The main effect analysis results are shown in Table 5. SI of village cadres has a significantly positive effect on IB of villagers (β = 0.488, *p* < 0.005) and AC (β = 0.721, *p* < 0.005). The AC has a significantly positive effect on IB of villagers (β = 0.689, *p* < 0.005). The above results support hypothesis H1. The statistical results of the data show that the SI of village cadres, such as providing advice (building a vision), shaping beliefs, and caring for the villagers, can enhance the sense of belonging of the villagers to villages and promote IB of the villagers.

**Table 5:** Main effect analysis.

**Path**	**Proposed assumptions**	**Standardization coefficient**	**SE**.	***R*^**2**^**	***Z*-value**	**Result**
SI → IB	+	0.488^***^	0.050	0.238	9.706	Support
SI → AC	+	0.721^***^	0.034	0.520	21.399	Support
AC → IB	+	0.689^***^	0.038	0.475	17.975	Support

*** *p < 0.001*.

This paper uses bootstrap technology to test the mediating effect of AC (the results are shown in Table 6) to test whether AC plays a mediating role in the positive impact of SI of village cadres on IB of villagers. Through the bootstrap analysis of 1,000 repeated samples, the results show that the mediating effect of AC (β = 0.507, SE = 0.050) is positively significant in the confidence interval without 0 (bias correction 95% CI is between 0.384 and 0.690, percentile 95% CI is between 0.368–0.661). The positive impact path of the village cadres on IB is realized through the mediating role of AC. The test results support hypothesis H2.

**Table 6:** The mediating effect of affective commitment.

**Effect**	**Standardization coefficient**	**SE**.	***Z*-values**	**Bootstrapping**			
				**Bias-Corrected 95% CI**		**Percentile 95% CI**	
				**Lower**	**Upper**	**Lower**	**Upper**
Total effect	0.498	0.050	10.000	0.358	0.635	0.354	0.629
Indirect effect	0.507	0.063	8.071	0.384	0.690	0.368	0.661
Direct effect	−0.010	0.080	−0.119	−0.155	0.152	−0.155	0.152

*The number of bootstrap samples is 1,000*.

### The Moderating Effect Test

To avoid multicollinearity among the regression model variables, we first centralized the interaction terms of SI, IB, resilience, AC, and the interaction between SI and resilience, AC, and resilience. On this basis, the hierarchical regression analysis was adopted to test the data. Step 1: Seven control variables, such as gender and education, were added to the regression analysis of IB. Step 2: SI and resilience were added to Path B. AC and resilience were added in Path B. Step 3: The interaction between SI and resilience was added in Path A. The interaction between AC and resilience was added in Path B. Table 7 shows the results of the moderating effect of resilience; the interaction between mental stimulation and resilience is 0 (95% CI is between −0.199 and −0.004), which indicates that the moderating effect of resilience on mental stimulation and IB is not significant. The test results do not support hypothesis H3. However, the interaction between AC and resilience did not include 0 (95% CI between −0.234 and −0.099), and the interaction was negative (β = −0.120, *p* < 0.05). The results show that resilience weakens positive impact of AC on innovation behavior only in the path relationship between AC and IB of villagers. This result is counterintuitive. Therefore, we further explore whether mediating effect of AC on IB of villagers will change at the high and low levels of resilience and change.

**Table 7:** Analysis results of the moderating effect of resilience of villagers.

**Step**	**Variables and models**		**Coefficient**	**SE**.	***P*-value**	**LLCI**	**ULCI**
The first step	Control variable	Gender	0.160	0.135	0.253	−0.099	0.418
		Age	0.181	0.165	0.272	−0.162	0.495
		Education	0.038	0.083	0.624	−0.115	0.218
		Number of children	−0.056	0.100	0.570	−0.232	0.173
		Party member	0.030	0.179	0.862	−0.324	0.392
		Cooperative member	−0.150	0.177	0.407	−0.497	0.211
		Marital status	0.043	0.172	0.797	−0.296	0.379
The second step	Path a	Independent variable: SI	0.276	0.061	0.001	0.155	0.399
		Moderator: Resilience	0.406	0.094	0.001	0.227	0.580
	Path b	Mediating variable: AC	0.489	0.060	0.001	0.352	0.591
		Moderator: Resilience	0.219	0.089	0.026	0.063	0.417
The third step	Moderating Effect a	SI × Resilience	−0.089	0.054	0.098	−0.199	0.004
	Moderating Effect b	AC × Resilience	−0.120	0.059	0.035	−0.234	−0.009

### Moderated-Mediating Effects Test

H4 proposed that the relationship between SI of village cadres and IB of villagers would be moderated by resilience through mediating transmission of AC. According to Edwards (2007), mediating effect of AC between SI and IB is analyzed when resilience is at different levels. Table 8 shows that, when resilience was at the high level, the mediating effect of AC on IB of villagers was positively significant (β = 0.376, *p* < 0.005); when resilience was at the low level, the mediating effect of AC on IB of villagers was also positively significant (β = 0.340, *p* < 0.005). The difference between the two groups was 0.036 (*P* < 0.005). The mediating effect of a high level of resilience is higher than that of a low level, which indicates that resilience positively regulates the mediating effect between SI and IB. Therefore, hypothesis H4 is verified.

**Table 8:** Analysis results of the moderated mediation model.

**Group and difference**	**SI (X)** **→** **AC (M)** **→** **IB (Y)**				
	**Stage**		**Effect**		
	**Stage 1 (P_**MX**_)**	**Stage 2 (P_**YM**_)**	**Direct effect (P_**YX**_)**	**Indirect effect (P_**MX**_P_**YM**_)**	**Total effect (P_**YX**_ + P_**MX**_P_**YM**_)**
High level of resilience (mean +1 standard deviation)	0.628^***^	0.598^**^	0.413^**^	0.376^***^	0.789^***^
Low level of resilience (mean −1 standard deviation)	0.580^***^	0.587^**^	0.384^***^	0.340^***^	0.724^**^
Difference between groups	0.048^**^	0.011^***^	0.029^***^	0.036^**^	0.065^**^

*P_MX_ refers to the path from SI to AC, and P_YM_ refers to the path of AC to IB. The direct effect P_YX_ refers to the path from SI to IB. The mediating effect refers to the product of the path coefficient between the first stage and the second stage. The total effect is the sum of direct effect and intermediary effect. The difference between groups is the difference between high-level resilience and low-level resilience*

*** *P < 0.001*;

** *P < 0.005*;

* *P < 0.01*.

## Discussion

Based on a questionnaire survey of 274 villagers in 23 provinces of China, our research uses the behavioral psychology experimental method to explore the internal mechanism of SI of village cadres on IB of villagers. The results show that the SI of the village cadres significantly impacts IB of villagers (the influence coefficient is 0.488). AC of villagers to the village mediates the influence of SI of village cadres on IB of villagers (the influence coefficient is 0.384). This mediating effect is positively moderated by the resilience of the villagers, which further affects SI of village cadres on IB of villagers. This indicates that attachment and AC of villagers to the villages play an essential role in enhancing the influence of SI of village cadres on IB of villagers. The more affectionate the villagers are, the more enthusiasm the village cadres can inspire. If the villagers are no longer attached to the village, no matter how motivated the village cadres are, the villagers will not engage in actions beneficial to the village. Moreover, the stronger the personalities of the villagers are, the more incentive the village cadres have to promote the IB of the villagers. If the personalities of the villagers are fragile and pessimistic, even if the villagers have an attachment to the village, the SI of the village cadres cannot promote the IB of the villagers.

We found the moderating role of personal psychological traits in the influence of leader motivation on IB of followers, and we confirmed that the extent to which the SI of the village cadres influences the innovation behavior of the villagers is moderated by the resilience of the villagers. Previous studies used moderating variables, including external team social capital (Deng et al., 2019), perception of organizational support (Giorgi et al., 2020), AC (Li, 2018), etc. Some of these factors refer to pure external forces; some refer to personal perception, but none of them involve personal psychological characteristics.

Our study also found that AC plays a mediating role in the influence of motivation on IB. In the previous literature on the influence of leader motivation on the IB of followers (employees), AC was been discussed as a moderator, but other possible effects were not studied (Li, 2018). Similar findings can be found in Kim and Park (2015), Li (2018), Qi et al. (2019), Giorgi et al. (2020), and Deng et al. (2019), who take, respectively, creative self-efficacy, innovative organizational culture, psychological empowerment, perception of organizational support, and team reflexivity as mediating variables. These variables are related to self-perception, and our research also confirms this point. Moreover, we confirm that the SI of the village cadres has a significant positive impact on the innovation behavior of the villagers, which is consistent with the conclusions of Li (2018), Qi et al. (2019), and Deng et al. (2019).

We propose the following three reasons for the significant positive impact of SI of village cadres on IB of villagers. First, the village cadres are the elite of the rural front-line administrators, the models and spiritual guides of the villagers (He and Akuzhiko, 2006). Second, the SI of the village cadres helps to generate AC of villagers to the village (Li, 2018). Third, the SI of the village cadres can help the villagers avoid the loss of innovation resources, increase the enthusiasm for innovation, promote the realization of innovation, and ultimately enhance the survival and development ability of the village (Bengt and Morten, 2000).

AC of villagers plays a mediating role in the influence of SI of village cadres on the IB of villagers. This may be because village cadres often motivate villagers through speeches, meetings, and public activities. These behaviors make villagers feel pride and love for their hometown, making villagers realize that they are members of the village and that they should contribute to the village. In this way, the villagers can have an emotional attachment and identity to the village organization, and increase their involvement level, their AC to the village. The AC of the villagers to the village encourages the villagers to contribute actively to the development of the village through innovation. The resilience of the villagers has a moderating effect on the impact of SI of village cadres on IB of villagers. This may be due to the realization that innovation needs meet some personal needs. The extent to which the SI of the village cadres can impact the IB of the villagers still depends on own characteristics and abilities of the villagers.

Although China has a vast territory and a large rural population, farmers in different places have high homogeneity. Specifically, our research is based on the background of the collective economy, clan culture, official culture, and the change of population structure in rural China. Among these factors, the collective economy is the economic system that covers all the rural areas of China, and the clan culture and the official and gentry culture have had a profound impact on all rural areas of China throughout history. In addition, the migration of rural population to cities is also a common situation faced in all the rural areas of China. Therefore, the duality of urban and rural governance and the administration of rural autonomy, which are directly caused or influenced by the above factors, are the same throughout China. We believe that the objects selected in this study are representative in the context of this study. Therefore, we can ensure the generalization of the research results.

There are three main limitations of this study. First, the sample size of this study is small, considering the huge amount of Chinese villagers. Second, the analyses were based on cross-sectional data, and there is no follow-up evaluation to verify the long-term stability of the theoretical framework. Third, there is no paired survey to eliminate common method bias, which may have led to inaccurate conclusions. We hope to alleviate the three limitations by expanding the sample and tracking investigation in further research.

## Conclusion

Based on survey research of 23 provinces in China, we studied the internal mechanism of inclusive innovation of bricolage. We found that SI of the village cadres is positive related to IB of villagers. Moreover, we also identified that AC of villagers to the village plays a mediating role in the relationship between the SI of the village cadres and the IB of the villagers. In addition, our findings reveal that resilience of villagers plays a moderating role in the relationship between SI of the village cadres and IB of villagers.

This paper discusses the relationship between village cadres and spiritual factors of villagers in rural areas, especially the positive effect of spiritual leadership of village cadres. Village cadres belong to a leading semiprofessional group of informal organization systems (villages) with Chinese characteristics. The internal logic and the mechanism of the relationship between village cadres and villagers are constructed accordingly. Since the agricultural tax was abolished entirely in 2006, rural governance and village development have been relying more on leadership of village cadres. The SI of village cadres is an essential source of innovative ideas and behaviors of villagers. Therefore, the quality, ability, and spiritual influence should be taken as critical bases for selecting cadres.

The research also reveals the mediating mechanism of AC in SI of leaders with IB of their subordinates, providing a theoretical framework to explain how leaders promote IB of subordinates. The importance of affection of villagers is highlighted. Therefore, it is imperative to make villagers feel secure by improving their living quality, enhancing their sense of happiness and achievement. To a certain extent, this confirms the significance of the vigorous efforts of the country for the construction of a beautiful village program. This study shows that encouragement of village cadres can stimulate the sentimental attachment of the villagers and their families, thereby increasing their IB. In addition, we reveal that the mediating role of AC of SI of leaders in IB of subordinates depends on resilience of villagers. The willpower, perseverance, and hard-working spirit of subordinates are inseparable, which guarantees the study of the benign operation effect of the model. The resilience may be either natural or cultivated. Therefore, it is necessary to give regular professional lectures to the villagers to develop this quality.

## Data Availability Statement

The original contributions presented in the study are included in the article/supplementary material, further inquiries can be directed to the corresponding author/s.

## Ethics Statement

The studies involving human participants were reviewed and approved by Ethics Committee of Guizhou University of Finance and Economic. Written informed consent for participation was not required for this study in accordance with the national legislation and the institutional requirements.

## Author Contributions

QZ provided the initial idea, built the initial theoretical framework, and led the data collection. MY participated in data collection, did data analysis, and wrote the main body of the article. In the process of reviewing the manuscript, LL provided suggestions on the structure and language modification of the article. BL collected and collated the literature, revised the theoretical framework, wrote the preface, discussion and conclusion, and led the revision in the process of manuscript review. LF completed the original translation. All authors contributed to the article and approved the submitted version.

## Conflict of Interest

The authors declare that the research was conducted in the absence of any commercial or financial relationships that could be construed as a potential conflict of interest.

## Publisher's Note

All claims expressed in this article are solely those of the authors and do not necessarily represent those of their affiliated organizations, or those of the publisher, the editors and the reviewers. Any product that may be evaluated in this article, or claim that may be made by its manufacturer, is not guaranteed or endorsed by the publisher.

## Appendix

**Appendix 1:** Construct and measurement.

**Variables**	**Construct**	**Items**	**Factor loading**	**Cronbach's α**	**References**
Independent variable	SI	1. I am confident in fulfilling the goals set by the village cadres.	0.827	0.948	Fry, 2003
		2. I always try my best to do a good job, because I have faith in the village collective leadership and the village cadres.	0.875		
		3. I set a high goal for my own work, because I am confident in the village collective leadership and hope that the villagers can succeed.	0.929		
		4. Our village collective leadership is trustworthy and always serves the villagers.	0.897		
		5. The village collective leadership really cares about the farmers.	0.898		
		6. The cadres in my village have the courage to stand up for their people.	0.78		
Mediator	AC	1. I am willing to work in the village in my spare time.	0.811	0.899	Meyer et al., 1993
		2. I take the village collective's issues as my own.	0.91		
		3. I have a strong sense of belonging to the village collective.	0.779		
		4. I want to be a member of the village collective.	0.825		
Moderator	Resilience	1. I can adapt to changes.	0.735	0.91	Campbell-Sills and Stein, 2007
		2. I can always stay optimistic when encountering difficulties.	0.836		
		3. I can continue to concentrate on my work under pressure.	0.866		
		4. I can cheer up after being sick, injured, or experiencing other difficulties.	0.863		
		5. Being under pressure makes me stronger.	0.734		
		6. I think I can achieve my goal even if there are obstacles.	0.739		
Dependent variable	IB	1. I will actively look for new technologies to improve labor efficiency	0.644	0.897	Scott and Bruce, 1994
		2. I will think about new ideas.	0.82		
		3. I will introduce my new ideas to others.	0.886		
		4. I will raise money for new ideas.	0.897		
		5. I will turn new ideas into reality.	0.752		
